# Exploring two-way text messages for post-discharge follow-up and quality improvement in rural Uganda

**DOI:** 10.1371/journal.pone.0322969

**Published:** 2025-08-11

**Authors:** Charly Huxford, Bella Hwang, Dustin Dunsmuir, Yashodani Pillay, Fredson Tusingwire, Florence Oyella Otim, Beatrice Akello, Aine Ivan Aye Ishebukara, Stefanie K. Novakowski, Bernard Opar Toliva, Nathan Kenya-Mugisha, Abner Tagoola, Matthew O. Wiens, Niranjan Kissoon, J. Mark Ansermino

**Affiliations:** 1 Department of Anesthesiology, Pharmacology & Therapeutics, University of British Columbia, Vancouver, British Columbia,; 2 Institute for Global Health, BC Children’s Hospital and BC Women’s Hospital + Health Centre, Vancouver, British Columbia,; 3 World Alliance for Lung and Intensive Care Medicine in Uganda, Kampala, Uganda; 4 Department of Pediatrics, Gulu Regional Referral Hospital, Gulu, Uganda; 5 Department of Pediatrics, Jinja Regional Referral Hospital, Jinja, Uganda; 6 Department of Pediatrics, University of British Columbia, Vancouver, British Columbia; University of Minnesota, UNITED STATES OF AMERICA

## Abstract

**Introduction:**

Automated messaging through text (SMS) and instant messaging services (IMS) offers low-cost solutions for patient follow-up in resource-constrained contexts. This study aims to evaluate a quality improvement (QI) initiative to improve caregiver response rates to an automated messaging system for post-discharge follow-up of children in rural Uganda.

**Methods:**

From June 2022 to June 2024, caregivers of children triaged through the Smart Triage platform at Gulu Regional Referral Hospital were invited to participate in an automated follow-up program. Messages were sent seven days after discharge via SMS and IMS (WhatsApp), prompting caregivers to report if their child had “improved” or “not improved”. Non-responders and “not improved” cases were followed up with a phone call from a study nurse. From April 2023 to November 2023, a QI initiative refined the messaging system to improve response rates and a post-QI period then continued the intervention with no changes until June 2024. Response rates were analyzed over three periods: historical (pre-QI, June 2022 – March 2023), QI intervention, and post-QI. Additionally, data on message delivery rates, improvement strategies, and health outcomes were analyzed.

**Results:**

Of 6826 participants, 6469 (95%) messages were successfully delivered. Response rates improved from 20% in April 2023 to 40% in June 2024 and remained stable between 33% and 41% during the post-QI period. Compared to the historical period, post-QI response rates were significantly higher (95% CI: 12.5% to 18.2%, p < 0.001). This improvement reflected a statistically significant positive trend during the QI period. Overall, 1856 caregivers responded: 1244 (67%) reported improvement and 612 (33%) reported no improvement. Follow-up phone calls for those “not improved” revealed 58 (9%) sought care, 12 (2%) were readmitted, and no deaths occurred. For non-responders, 206 (5%) sought care, 33 (0.7%) were readmitted, and 3 (0.07%) deaths occurred.

**Discussion:**

Automated two-way text messages for post-discharge pediatric follow-up yielded high delivery and moderate response rates. Iterative QI efforts increased response rates, highlighting the importance of tailored communication strategies. Automated messages can facilitate timely intervention for high-risk children and enable efficient collection of health outcomes, offering a viable alternative to in-person follow-up in resource-poor settings.

## Introduction

Post-discharge mortality often exceeds in-hospital rates in low-resource settings [1]. The causes of post-discharge deaths are multi-factorial and include poverty, inability to access timely care, non-resilient health systems, and poor follow-up after discharge. Follow-up of all children post-discharge may not be feasible or even necessary; however, it is certainly a need among those at high risk for post-discharge death. In many cases in-person follow-up is resource intensive and impractical and hence largely ignored or poorly conducted, leaving patients and families with little support during the vulnerable post-discharge period. Our group developed, validated, implemented, and evaluated a digital triage platform called Smart Triage [2–6] to prioritize initial emergencies at facility level. Leveraging data from this platform, we sought to explore ways of improving communication and follow-up after discharge using Short Message Service (SMS) and Instant Messaging Service (IMS) communication.

Previous studies have demonstrated the usability and acceptability of SMS-based interventions, and their potential to enhance treatment adherence and healthcare communication in low-resource settings; however, their effectiveness for structured post-discharge follow-up in pediatric populations remains largely unexamined [7,8]. Mobile phone penetration has risen dramatically in sub-Saharan Africa, with an estimated 49% of the population owning a smartphone and 82% having access to basic mobile phones [9]. SMS and WhatsApp were selected for facilitating follow-up due to their widespread availability and frequent use in daily life. In Uganda, texting is the most common activity among mobile phone users [10]. These messaging platforms offer low-cost, scalable solutions for engaging caregivers, alleviating the burden on overextended health workers, and collecting actionable data in real time. However, limited access to technology among the lowest income households, varying literacy levels, and inconsistent network coverage remain barriers to using text messaging for post-discharge follow-up in resource-poor settings. Furthermore, cultural and contextual factors may influence caregiver engagement and response rates, necessitating a tailored approach to ensure successful implementation [11].

This study aimed to evaluate the implementation of a quality improvement (QI) intervention to increase response rates to automated two-way text message follow-ups for children discharged from Gulu Regional Referral Hospital (GRRH) in Northern Uganda. Additionally, the study explored the potential of using these text messages to support real-world outcome monitoring of a pediatric clinical prediction model and improve communication between caregivers and health workers.

## Methods

### Study design and setting

The study was conducted in the outpatient department (OPD) of GRRH over a two-year period, from June 23, 2022, to June 30, 2024. GRRH is a public hospital funded by the Uganda Ministry of Health and its OPD serves as a primary healthcare provider for over 23,000 pediatric and adult patients annually, offering free services to residents of five districts in Northern Uganda: Amuru, Gulu, Kitgum, Lamwo, and Pader.

The follow-up intervention augments the Smart Triage platform, a pediatric digital triage system that uses a data-driven risk prediction model and treatment tracking system for children under 18 years old [2–6]. Developed over the past decade, Smart Triage aims to collaboratively develop and implement clinically validated tools to allocate resources more efficiently in low-resource contexts and improve outcomes for children with an acute illness upon arrival to outpatient (emergency) departments. Initially, all caregivers were enrolled during triage for telephone follow-up with a study nurse seven days after discharge. The follow-up phone calls were supplemented with the automated two-way text messaging system in June 2022.

### The intervention

#### Automated follow-ups.

The automated two-way follow-up aimed to assess the child’s health status seven days after discharge, either after the child was sent home directly from outpatient (emergency) care or following an inpatient stay. At triage, caregivers were offered the option to receive an automated follow-up message. Those who agreed selected their preferred communication channel—SMS or WhatsApp—and preferred language. SMS messages were available in English or Acholi, while WhatsApp messages were limited to English. The follow-up message asked caregivers for a simple binary response at no cost to them: *“A health worker from Gulu Hospital is following up with you to see if your child [child’s name] has improved. Please reply with ‘1’ if your child has improved or ‘2’ if your child has not improved. This number is toll-free. Send STOP or 196 to unsubscribe.”*

The automated follow-up was used to immediately identify children who had not improved. Caregivers who responded that their child had not improved were flagged for further follow-up by a study nurse, who contacted them by phone as soon as possible. A reminder message was automatically sent if caregivers did not respond within three days of receiving the initial message. Non-responders (those who did not reply within seven days), caregivers who unsubscribed (those who responded with STOP or the number 196), and caregivers who sent an invalid response (a text-based response other than 1 or 2) were also contacted by phone to assess the child’s status. Those who responded that their child had improved were sent an automated acknowledgment message, with no further follow-up. Caregivers who were unreachable by phone call after multiple attempts were considered lost to follow-up.

#### Automated follow-up system technology.

Data included follow-up dates, phone numbers, responses to the messages, and responses to the phone call questions and were stored in a Research Electronic Data Capture (REDCap) database. REDCap is a secure and encrypted, web-based application designed for data collection and management in research studies and QI projects [12]. REDCap required 2-factor authentication to access, which maintained the security of caregiver and patient contact information. The automated follow-up system used Africa’s Talking (https://africastalking.com) for SMS and Twilio (https://www.twilio.com/en-us) for WhatsApp messaging. Seven days after discharge, messages were sent through the caregiver’s preferred channel—SMS or WhatsApp—using a scheduled server process that triggered message delivery daily at 4:00 PM (originally 9:00 AM) East Africa Time. Caregiver responses to messages were processed by a dedicated callback URL which logged and updated REDCap. Automatic logging and daily monitoring by the study team ensured messages were sent and delivered. Responses were automatically tracked within REDCap. Issues with message delivery, such as failed WhatsApp messages, were also recorded automatically on REDCap. Failed WhatsApp messages were automatically resent as an SMS message.

#### SMS.

Africa’s Talking was used for SMS follow-ups through a dedicated short code (4-digit phone number available in Uganda). The SMS delivery server application used the Africa’s Talking Python library (version 1.2.7) and caregiver responses were managed through callbacks to a Flask-based Application Programming Interface (API) that then updated the REDCap database.

#### IMS.

Caregivers who selected WhatsApp as their preferred communication method received automated messages via Twilio. Twilio required pre-approval for message text, which limited the ability to offer WhatsApp messages in Acholi. The local language, Acholi, has a relatively small number of native speakers and was not an included language on the Twilio platform. The Twilio Python server-side software development kit (SDK) was used to initiate conversations based on REDCap data and then a flow that was created in Twilio Studio controlled the conversation logic and handled responses, including updating to the REDCap research database.

#### QI to increase the message response rate.

**Fig 1:**
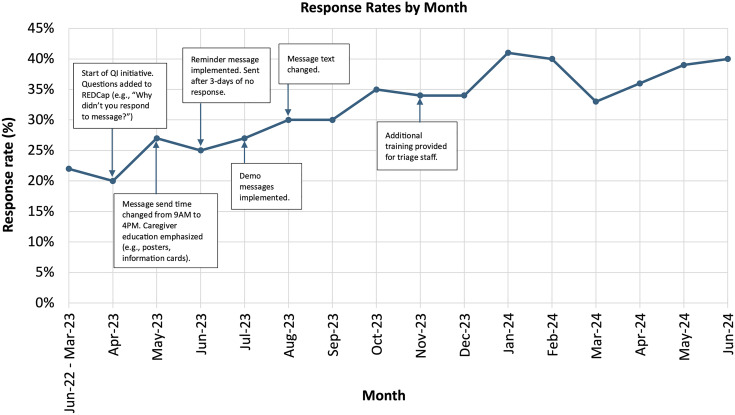
Response rate change by month.

The baseline response rate was established during the historical period (June 2022–March 2023), prior to the implementation of any QI changes. Beginning in April 2023, multiple iterative strategies were implemented on a monthly basis to improve response rates and refine the messaging process (Fig 1 and S1 Table). Response rates were monitored over time to assess the impact of each intervention, with adjustments made based on feedback and evolving needs to optimize follow-up engagement. For the purpose of analysis, the study period was divided into three phases: a historical period (June 2022–March 2023), a QI intervention period (April–November 2023), and a post-QI period (December 2023–June 2024). The QI period corresponded to the time during which iterative improvements to the follow-up system were actively implemented. The post-QI period was defined to assess whether improvements in response rates were sustained after the conclusion of active QI activities.

### Data collection

Responses to the automated messages were automatically recorded in REDCap in real-time, ensuring accurate and timely data entry. If a caregiver responded with “1” (improved) the case was marked as complete and the caregiver was sent an acknowledgment. Responses of “2” (not improved) and non-responses were tracked in a REDCap report, which was monitored daily. The study nurses attempted to call all caregivers in this report. During the phone call, caregivers were asked a standardized set of questions about their child’s health and the study nurses manually entered the responses into REDCap immediately following the interaction.

### Outcome measures

#### Delivery rate.

The success of the automated follow-up message is described by the delivery and response rates as percentages. Message send attempts may fail to be delivered due to a telecom outage, the number being not in service, or, for WhatsApp, the number not being connected to an account. Delivery rate was defined as the percentage of successfully delivered messages out of all messages sent.

#### Response rate.

The response rate was defined as the percentage of messages replied to out of the total number of messages delivered. A response was defined as the caregiver responding that their child had improved or not improved. During the follow-up phone calls, caregivers who did not respond to the text messages were asked for their reasons, which were broadly categorized into technology-related issues and caregiver-related factors. Reasons for non-response could not be obtained if the caregiver was unreachable, if they claimed to have replied but no response was recorded, or if no specific reason was provided.

#### Health outcomes.

Health outcomes were collected with a standardized set of questions asked by the study nurse during the phone call follow-up. The questions about their child’s condition included whether they had sought additional medical care without readmission, if the child had been readmitted, and if the child had died. The collected data were analyzed to determine the proportion of children experiencing each outcome.

#### Statistical analysis.

Data analysis was completed using Excel (version 16.97.2) and R (version 4.2.2). A post-hoc Cochran-Armitage trend test was performed to assess for a statistically significant trend in response rates during the QI intervention period (April–November 2023), with the Z value and p-value reported (S2 Table). A post-hoc two-proportion z-test was conducted to compare overall response rates between the historical period (June 2022–March 2023) and the post-QI period (December 2023–June 2024). For this comparison, we reported the observed difference in proportions, 95% confidence interval, p-value, and results of a post-hoc power calculation estimating both the required sample size to achieve 80% power (alpha = 0.05) and the actual achieved power based on the observed effect and sample sizes (S2 Table).

### Ethics

Ethics approval for this study was obtained from the institutional review boards at Makerere University School of Public Health in Uganda (SPH-2021–41) and the Uganda National Council for Science and Technology (HS1745ES). As this is a QI project, patient consent was waived. Ethics approval for this study was not required by the University of British Columbia’s (UBC) Research Ethics Board. UBC adheres to the Canadian government’s Tri-Council Policy 2 (TCPS2) Statement which states that quality assurance and quality improvement (QA/QI) studies, program evaluation activities, and performance reviews, or testing within normal educational requirements, when used exclusively for assessment, management or improvement purposes, do not constitute research under the TCPS2 and do not fall under the scope of REB review. See section 4.4.1 of the UBC Clinical Research Ethics General Guidance Notes. No data was collected in Canada. This QI project was reported according to the Standards for Quality Improvement Reporting Excellence (SQUIRE) 2.0 guidelines [13]. Additional information regarding the ethical, cultural, and scientific considerations specific to inclusivity in global research is included in the Supporting Information (S3 File).

## Results

### Study population

During the study period, 6,826 children triaged through the Smart Triage platform were enrolled in the automated follow-up system. The median age was 1.4 years (IQR: 0.6 to 3.5 years), with 84% (5731) of children under 5 years of age. Overall, 54% (3673) of participants were male. The most common presenting complaints at triage were cough and/or respiratory illness (2732, 40%), diarrhea and/or vomiting (1505, 22%), and fever (1034, 15%). In terms of triage acuity, 52% (3531) of children were classified as non-urgent, 36% (2450) as priority, and 12% (845) as emergency cases at initial triage (Table 1).

**Table 1:** Characteristics of the study population, including age, sex, presenting complaint, and triage category at initial presentation. In cases where multiple presenting complaints were listed, the most severe complaint was selected for this table (e.g., convulsions were selected if cough, diarrhea, convulsions were all listed).

Characteristic	N = 6826 n (%)
Age, years (median)	1.4 (IQR 0.6–3.5)
Age categories	
0-11 months	2637 (39)
1-4 years	3094 (45)
5-9 years	1060 (16)
10-18 years	35 (0.5)
Sex	
Female	3153 (46)
Male	3673 (54)
Triage category	
Non-urgent	3531 (52)
Priority	2450 (36)
Emergency	845 (12)
Presenting complaint	
Cough and/or Respiratory Illness	2732 (40)
Diarrhea and/or Vomiting	1505 (22)
Fever	1034 (15)
Pain/Swelling	486 (7)
Skin Infections/Rash	372 (5)
Injury or Trauma	57 (0.8)
Difficulty Breathing	39 (0.6)
Seizures/Convulsions	38 (0.6)
Other^a^	563 (8)

^a^Other includes ENT/eye complaints, urinary complaints, irritable, oedema, etc.

### Delivery rate

**Fig 2:**
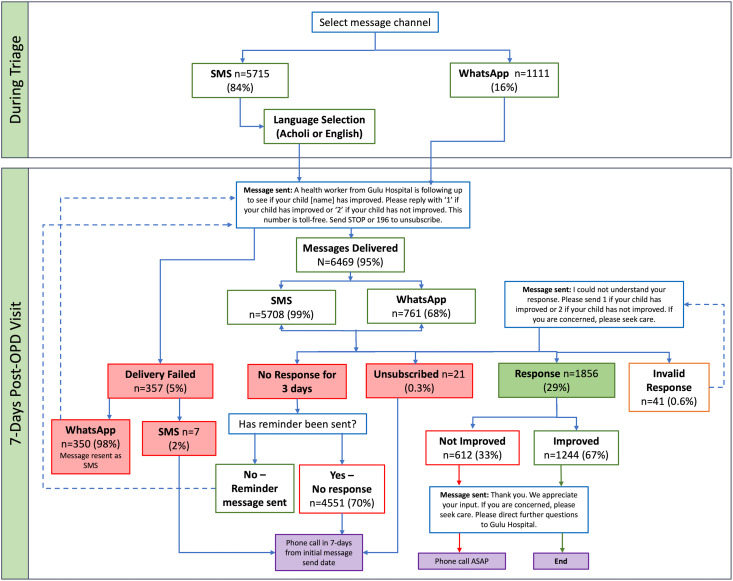
Messages sent and received.

Among enrolled participants, 84% (5715/6826) of caregivers chose SMS as their preferred channel and 16% (1111/6826) chose WhatsApp (Fig 2). The overall delivery rate was 95%. Of the 357 (5%) undelivered messages, most (98%) were WhatsApp messages, while 2% were SMS messages.

### Response rate

Between June 2022 and March 2023, before the implementation of any QI changes, the response rate was 22% (507/2292). Response rates improved from 20% in April 2023 to 40% in June 2024, following the introduction of iterative QI changes. During the post-QI period (December 2023 – June 2024), response rates remained stable between 33% and 41%, indicating sustained improvements beyond the active intervention phase. Compared to the historical period (June 2022 – March 2023), the overall response rate during the post-QI period was significantly higher (observed difference: 15.4%; 95% CI: 12.5% to 18.2%, p < 0.001, S2 Table). The Cochran-Armitage trend test also demonstrated a statistically significant positive trend in response rates during the QI intervention period (April 2023 – November 2023) (Z = 5.32, p < 0.001; S2 Table). Between June 2022 and June 2024, 1856 responses were received. Out of those, 1244 (67%) caregivers responded that their child had improved, and 612 (33%) responded that their child had not improved.

The QI changes implemented were informed by the feedback from caregivers (Table 2).

**Table 2:** Reasons for not responding.

Reason participant did not respond	N = 1524 n (%)
Technical issue (e.g., broken phone)	588 (39)
Did not know how to reply to the message	267 (18)
Busy	230 (15)
Forgot to respond	223 (15)
Worried about the cost to respond	128 (8)
Did not know who was sending the message	26 (2)
Not interested	15 (1)
Could not be reached at number/is no longer available (caregiver provided number of relative, neighbor, friend, etc.)	6 (0.4)
Wrong number (caregiver gave wrong number or recipient does not know the caregiver)	5 (0.3)
Other^a^	36 (2)

^a^Other includes reasons such as not having data, phone was off, didn’t take it seriously, lost phone, didn’t check messages

The data gathered through follow-up calls with non-responders identified key barriers such as message delivery times, confusion about the sender, and lack of understanding of how to respond. For example, follow-up messages were initially sent at 9:00 AM. After receiving feedback that caregivers were often busy in the mornings, the message time was shifted to 4:00 PM to better align with their availability. Another strategy was to add a reminder message if no response was received three days after the initial message, to prompt caregivers who may have forgotten or missed the first message. In addition, to ensure caregivers understood how to respond, a demo message was sent to the caregiver’s phone at the time of their in-person triage. A health worker demonstrated and explained the follow-up message to caregivers. Additionally, caregiver education was enhanced using posters displayed in the OPD and information cards distributed during triage. The posters and cards provided additional information regarding the automated follow-up system, including the definitions of the response options and understanding the response process.

### Health outcomes

A total of 5582 caregivers were scheduled for follow-up phone calls by the study nurse. Those scheduled included 4551 caregivers (82%) who did not respond to the automated message, 612 (11%) who replied that their child had not improved, 41 (0.7%) who sent an invalid response, 21 (0.4%) who unsubscribed, and 357 (6%) for whom the message delivery failed (Fig 2). As part of the protocol, caregivers who unsubscribed from the messages were still followed up with by phone call. A total of 5619 caregivers were actually reached by phone, including 177 caregivers (3%) who received a follow-up phone call even though they responded that their child had improved (calls were not required for this group). This occurred due to either delayed caregiver responses, which arrived after the follow-up call had already been made, or errors by the study nurse. In total, 140 caregivers (3%) were lost to follow-up.

Among the 4551 non-responders, 206 caregivers (5%) reported seeking care, 33 (0.7%) stated their child had been readmitted, and 3 children (0.07%) had died by the time of follow-up (Table 3). Among the 612 caregivers who reported that their child had not improved, 58 (9%) indicated they had sought some care that did not lead to an improvement, 12 (2%) reported that their child had been readmitted, and none had died by the time of the phone call. The most common reasons for readmission were malaria (5 cases, 41%) and upper respiratory tract infections (URTI; 2 cases, 17%).

**Table 3:** Health outcomes collected during follow-up phone calls.

Outcome	Response (“not improved”) n (%) N = 612	No response n (%) N = 4551
Triage category		
Non-urgent	376 (61)	2317 (51)
Priority	179 (29)	1651 (36)
Emergency	57 (9)	583 (13)
Sought additional care (not readmitted)	58 (9)	206 (5)
Non-urgent	32 (55)	76 (37)
Priority	16 (28)	65 (32)
Emergency	10 (17)	65 (32)
Readmission	12 (2)	33 (0.7)
Non-urgent	3 (25)	7 (21)
Priority	6 (50)	9 (27)
Emergency	3 (25)	17 (52)
Readmission reason		
Malaria	5 (41)	10 (30)
URTI (cold, flu, etc.)	2 (17)	1 (3)
Anemia	1 (8)	2 (6)
Meningitis/other CNS	1 (8)	0
Sepsis	0	6 (18)
Pneumonia	0	6 (18)
Gastroenteritis/Diarrhea	0	3 (9)
Other	3 (25)	5 (15)
Death	0	3 (0.07)
Non-urgent	0	0
Priority	0	2 (67)
Emergency	0	1 (33)
Data unavailable		
Lost to follow-up	7 (1)	124 (3)

## Discussion

The automated text messaging system achieved a high delivery rate (95%), with the majority of undelivered messages (98%) sent via WhatsApp. Response rates improved following iterative strategies based on caregiver feedback which helped refine the intervention and address real-world barriers to engagement. Among the iterative changes implemented during the QI period, the combination of adjusting the message send time to 4:00 PM and introducing caregiver education materials in May 2023 had the most substantial immediate impact on response rates. This change resulted in an increase from 20% in April to 27% in May. Smaller but meaningful increases were also observed following the simplification of message text in August 2023. Overall, the cumulative impact of these targeted modifications contributed to a sustained improvement in engagement throughout the QI and post-QI periods. Strategies to address non-response to follow-up messages should consider context, available resources, and risk assessment. Our findings suggest that non-responders may have experienced fewer adverse outcomes than those who responded, but this observation is based on descriptive data and should be interpreted with caution. It is possible that failure to respond reflects caregiver perception of improvement in the child’s condition, though future research using more robust analyses is needed to explore this. A selective approach to following up with non-responders that prioritizes those identified as high-risk during hospital triage or focuses efforts where there is a higher likelihood of adverse outcomes may be reasonable, although further research would be needed to assess such an approach. Such a strategy would balance resource constraints with the need for effective care, allowing for regionally and contextually appropriate interventions tailored to what is affordable and achievable within the local healthcare infrastructure.

We used SMS and IMS as simple and effective solutions for post-discharge follow-up in children. Each solution has advantages that will evolve with advances in technology availability. SMS is more widely available and is supported by feature phones but requires a relay service. IMS requires a smartphone and data plan, but it is more reliable as it does not involve the local telecom and can be delivered at a lower cost and at scale. By combining an automated two-way messaging system with an iterative QI approach, we systematically addressed barriers to caregiver engagement and achieved sustained improvements in response rates over the study period. While the peak response rate of 40% may appear modest, it is important to note that this encompassed all children who were triaged, rather than focusing solely on those at higher risk. A 100% response rate may be unrealistic when enrolling all discharged children due to systemic barriers such as limited mobile network coverage and caregiver concerns about the cost of responding. The messaging system could potentially be combined with a risk prediction model to help identify non-responders most in need of a follow-up call by a human. Such an approach might help reduce the impact of low response rates by distinguishing between children likely to be well and those who may require further care, including caregivers who should have sought medical care for their children but did not.

### Comparison with other studies

SMS-based interventions are effective in healthcare settings in low-and middle-income countries (LMICs) and are reported to improve childhood immunization coverage and timeliness [11], increase attendance at maternal and neonatal healthcare visits [14], and support treatment adherence in the management of hypertension and HIV [15, 7]. However, many studies using text messages have primarily focused on sending reminders or behavior change interventions aimed at improving specific outcomes, such as appointment attendance and medication adherence, and mostly in non-urgent conditions where there is time for deliberation [16].

Strategies to improve response rates for text-based interventions include personalizing messages, such as incorporating the patient’s name or tailoring content to specific health conditions [17, 18]. Sending messages at times aligned with recipients’ routines or preferences also improves response rates [17, 18]. In our study, strategies such as personalizing messages to include the child’s name and adjusting the timing of message delivery were implemented, leading to an overall increase in response rates.

The potential for text-based interventions to improve post-discharge outcomes is highlighted by a study conducted in rural Kenya [19]. The study evaluated the use of SMS for following up with caregivers of children after discharge. The findings revealed improvements in caregiver knowledge, increased efficacy in childcare, strengthened relationships with healthcare staff, and reduced hospital readmission rates [19].

### Strengths and limitations

The strength of our study is the iterative QI approach which tailored the system for the environment in which it was routinely used. We drew on successful strategies from previous studies, including the WelTel Kenya1 study [7], which demonstrated the effectiveness of using simple messages such as “How are you?” to encourage responses and enable self-reporting of problems. Building on this approach, our system utilized two-way text messaging to confirm whether caregivers received and engaged with the messages and to capture valuable feedback on the health of children once they returned to their community. This feedback may allow health workers to respond quickly when caregivers report a lack of improvement in their child’s condition, potentially preventing adverse outcomes.

There is a critical need for community post-discharge outcome data and follow-up care for high-risk children, as well as a more efficient approach to reduce the burden on health workers and healthcare resources. Before the introduction of the automated follow-up system, health workers attempted to call every caregiver of a discharged child—a highly resource-intensive process, with each call taking at least five minutes. A more targeted approach, in which follow-up calls focus only on caregivers who do not respond to messages or report that their child has not improved, can streamline efforts and make follow-up more feasible. While individual phone calls remain time-intensive, prioritizing high-risk cases using data-driven strategies can reduce overall resource demands compared to traditional methods. Additionally, the system ensures timely data collection, which is essential for driving QI initiatives and refining clinical risk prediction models. The growing response rates, along with the simplicity and accessibility of text messaging, suggests SMS/WhatsApp messaging could be a viable alternative to traditional follow-up methods, such as return visits, particularly in settings with limited infrastructure and long travel distances. Beyond facilitating follow-up, the system captures valuable community-level outcomes, such as readmissions and post-discharge mortality, providing crucial data for clinical and public health planning. Furthermore, the collected data may help identify key trends, including seasonal variations in disease prevalence and shifts in care-seeking behaviors, which can inform targeted public health interventions.

Limitations are related to the technology and barriers faced by caregivers. Undelivered messages due to rurality and infrastructure (e.g., caregivers lacking active accounts or compatible devices, inconsistent mobile network coverage, and occasionally disrupted message delivery) were significant, given the total number of messages sent. Caregiver-related barriers included difficulty understanding the messages, competing priorities, unawareness of the sender or purpose of the message, and concerns about cost (although they were assured that this is not an issue). Incorrect or outdated phone numbers and broken screens and keypads were also problems. These findings highlight the importance of addressing behavioural and technological obstacles to improve response rates.

The costs associated with this system are relatively high in the Ugandan context, with fixed monthly and annual payments to Africa’s Talking and the Ugandan Communications Commission amounting to USD $3,776.00 per year. These costs are fees paid to the mobile network operators and for the use of a dedicated short code. Although the cost per individual message was low— USD $0.016 for outgoing WhatsApp and USD $0.0018 for incoming and outgoing SMS—these expenses can be costly at scale and accumulate significantly with high patient volumes. Twilio charges a small per-message fee for each outgoing WhatsApp message, which decreases as the volume of messages increases. However, the system could be cost-effective if it is shared across multiple facilities, distributing the fixed costs more efficiently. Mobile applications like WhatsApp offer a more cost-effective solution for communication compared to SMS. However, in our study, most caregivers preferred SMS as their communication channel. This preference may be attributed to the study’s setting in a relatively poor region of Uganda, where access to smartphones is likely limited for many caregivers. In the Acholi region which encompasses Gulu district, 68% of households owned a mobile phone compared to 93% in higher income areas like Kampala [20]. Despite these costs, the system has the potential to enable efficient care and reduce hospitalizations by improving follow-up, but its cost-effectiveness and scalability remain to be formally assessed. Leveraging predictive models [3, 4] to identify and target follow-ups for the most vulnerable children could maximize the impact of an automated follow-up system while minimizing unnecessary expenditures and could be a valuable investment in resource-constrained settings.

Additionally, several limitations are associated with the study design and analysis. The observational design without a comparator or control group limits the ability to draw causal inferences about the effectiveness of the interventions. While we observed changes over time, we cannot rule out the influence of external factors. The study was a quality improvement initiative and was not designed for formal hypothesis testing. Therefore, we primarily used descriptive statistics to track trends over time and guide adaptations to the intervention. While additional post-hoc statistical analyses were conducted to examine changes in response rates, these were not pre-specified and should be interpreted cautiously. In particular, the designation of a post-QI period was applied retrospectively to assess whether improvements were sustained. Smaller sample sizes and variable trends across other periods and months would have limited the ability to detect statistically significant differences. We found that this comparison between the historical period and the post-QI period was well-powered and showed a significant difference, but the findings should be interpreted as indicative of implementation trends rather than definitive proof of intervention effectiveness. These limitations reflect the real-world, pragmatic nature of the project. Future studies using more rigorous designs and statistical methods would be valuable to further evaluate this approach.

While mobile messaging can support post-discharge follow-up, it also raises ethical concerns. Not all caregivers had reliable access to phones, mobile networks, or the digital literacy needed to engage with the system. This may have unintentionally excluded some families and deepened existing health inequities. Although we prioritized caregiver privacy, digital follow-up still requires the collection of personal information. Even when anonymized, this raises important issues about consent, data ownership, and autonomy, particularly in low-resource settings where power imbalances may exist between families and the healthcare system. Future implementation should focus on improving digital access, clearly communicating how data will be used, and involving caregivers in the design and oversight of such systems.

## Conclusion

Automated two-way text messaging systems for post-discharge follow-up in children using SMS and WhatsApp to connect healthcare workers with caregivers is feasible. This study highlights the potential of integrating digital health technologies in resource-limited settings where traditional follow-up methods may be impractical or overly resource-intensive. While the system was able to identify children who had not improved after discharge, challenges with message delivery, non-response, and technological access remain. These findings demonstrate the practical potential and limitations of using simple digital tools to support follow-up care. Continued efforts to optimize response rates, address technological limitations, and improve accessibility are essential to maximizing the potential of digital health interventions for enhancing follow-up care. Work is ongoing with the Uganda Ministry of Health to integrate this program within their system.

## Supporting information

S1 Table Response rates over time with QI changes. Descriptive statistics showing the number of messages delivered, number of responses, and monthly response rates.
(DOCX)

S2 Table Summary of post-hoc statistical analyses of response rates. A Cochran-Armitage trend test was used to assess the presence of a statistically significant trend in monthly response rates during the QI intervention period (April–November 2023). A two-proportion z-test was used to compare overall response rates between the historical period (June 2022–March 2023) and the post-QI period (December 2023–June 2024). For this comparison, a power calculation was conducted to estimate the required sample size to achieve 80% power (alpha = 0.05) and to assess the actual achieved power based on the observed effect size and sample sizes.
(DOCX)

S3 File Inclusivity in global research. (DOCX)
