# Supplementary material for: Exploring two-way text messages for post-discharge follow-up and quality improvement in rural Uganda
Source: PLoS One. 2025 Aug 11;20(8):e0322969. doi: 10.1371/journal.pone.0322969 (PMC12338838; doi:10.1371/journal.pone.0322969)
Supplement: S1 Table — Descriptive statistics showing the number of messages delivered, number of responses, and monthly response rates. (DOCX) [file pone.0322969.s001.docx]

| Date Range | Changes Made | Messages Delivered (n) | Total Responses (n) | Overall Response Rate (%) |
| --- | --- | --- | --- | --- |
| *Historical Period: Pre-QI, before any iterative changes were made* | | | | |
| June 2022 – March 2023 |  | 2292 | 507 | 22% |
| *QI Period: Overview with iterative changes to the project (by month)* | | | | |
| April 2023 | Question added to REDCap for follow-up call (“Why did you not respond to the message?”). | 402 | 79 | 20% |
| May 2023 | Message send time changed from 9:00 AM to 4:00 PM.  Caregiver education emphasized (e.g., informational posters displayed in triage area, information cards handed out to caregivers). | 394 | 107 | 27% |
| June 2023 | Reminder message implemented to be sent 3 days after no response. | 259 | 66 | 25% |
| July 2023 | Demo message implemented to be sent during triage. | 177 | 48 | 27% |
| August 2023 | Message text changed to be simpler. | 210 | 63 | 30% |
| September 2023 – October 2023 | No changes. | 518 | 170 | 33% |
| November 2023 | Additional training provided for triage staff on the automated message system and caregiver education. | 496 | 171 | 34% |
| *Post-QI Period: Sustainment after iterative changes made* | | | | |
| December 2023 – June 2024 | No changes. | 1721 | 645 | 37% |
