# Supplementary material for: Exploring two-way text messages for post-discharge follow-up and quality improvement in rural Uganda
Source: PLoS One. 2025 Aug 11;20(8):e0322969. doi: 10.1371/journal.pone.0322969 (PMC12338838; doi:10.1371/journal.pone.0322969)
Supplement: S2 Table — A Cochran-Armitage trend test was used to assess the presence of a statistically significant trend in monthly response rates during the QI intervention period (April–November 2023). A two-proportion z-test was used to compare overall response rates between the historical period (June 2022–March 2023) and the post-QI period (December 2023–June 2024). For this comparison, a power calculation was conducted to estimate the required sample size to achieve 80% power (alpha = 0.05) and to assess the actual achieved power based on the observed effect size and sample sizes. (DOCX) [file pone.0322969.s002.docx]

| Period | Time Frame | Overall Response Rate (%) | Observed Difference (%) | Statistical Test | Result | p-value | Required n | Actual n | Actual Achieved Power (%) |
| --- | --- | --- | --- | --- | --- | --- | --- | --- | --- |
| Historical (pre-QI) | June 2022 – March 2023 | 507/2292 (22) | - | - | - | - | - | - | - |
| QI Period | April 2023 – November 2023 | Increasing (month-to-month)  702/2456 (29) | - | Cochran-Armitage Trend Test | Z = 5.32 | p < 0.001 | - | - | - |
| Post-QI Period | December 2023 – June 2024 | 645/1721 (37) | - | - | - | - | - | - | - |
| Comparison: Historical vs. Post-QI | June 2022 – March 2023 vs. December 2023 – June 2024 | - | 15.4% | Two-Proportion Z-Test | 95% CI: 12.5% to 18.2% | p < 0.001 | 290 | 4013 | 100% |
